# Safety and efficacy of add-on intra-arterial thrombolysis after intravenous thrombolysis and mechanical thrombectomy in patients with ischemic stroke and cerebral vessel occlusion

**DOI:** 10.3389/fneur.2025.1560045

**Published:** 2025-05-30

**Authors:** Norma J. Diel, Kai Bernhard Woelk, Anne Mrochen, Oliver Posner, Andre Worm, Omar Alhaj Omar, Christian Claudi, Patrick Schramm, Tobias Struffert, Hagen B. Huttner

**Affiliations:** ^1^Department of Neurology, Justus-Liebig-University Hospital Giessen, Giessen, Germany; ^2^Department of Neuroradiology, Justus-Liebig-University Hospital Giessen, Giessen, Germany

**Keywords:** acute ischemic stroke, intravenous thrombolysis, endovascular treatment, intraarterial thrombolysis, mechanical thrombectomy

## Abstract

**Introduction:**

For acute ischemic stroke (AIS) with large vessel occlusion (LVO), the currently established treatment strategy of combined intravenous thrombolysis (IVT) and endovascular thrombectomy (EVT) is not sufficiently effective in all patients. Intra-arterial thrombolysis (IAT) as an adjunct to IVT/EVT may improve outcomes but may also increase the rate of hemorrhagic complications.

**Methods:**

This observational study analyzed data from the Giessen Stroke Registry (GIST; NCT05295862) between May 2022 and June 2024. Patients with AIS and LVO who received both IVT and EVT were included. A subset of patients received additional IAT (triple treatment, TT). Using 1:1 propensity score matching, 33 TT patients were compared with 33 controls who received only IVT + EVT. Primary outcomes were hemorrhagic complications (ECASS classification), and secondary outcomes included reperfusion rates, ASPECTS scores, 7-day mortality, and functional outcomes.

**Results:**

Baseline characteristics were balanced between the TT and the control group. The primary outcome was not significantly different with a rate of hemorrhagic complications of 3/33 (9%) in the TT group and 4/33 (12%) in the control group (OR 0.725, 95% CI 0.149–3.525). Secondary outcomes showed no significant differences with respect to rates of successful reperfusion, ASPECTS scores or 7-day mortality rates between TT and the control group.

**Conclusion:**

Triple treatment (IVT, EVT, and IAT) did not significantly improve clinical outcomes compared to IVT and EVT alone. However, TT was safe without signs of increased bleeding complications. TT should not be routinely used until further evidence verifies safety and substantiates a possible benefit in specific patient populations.

## 1 Introduction

Acute ischemic stroke (AIS), particularly in patients with cerebral vessel occlusion, is one of the leading causes of morbidity and mortality worldwide. Intravenous thrombolysis (IVT) and endovascular thrombectomy (EVT) have become established and highly effective treatment strategies for these patients. However, even with these interventions, sufficient recanalization of the affected brain tissue is only achieved in a subset of patients, and full recanalization remains an essential prognostic factor for better outcomes (1).

One proposed approach to augment treatment is the use of intra-arterial thrombolysis (IAT) as an adjunct therapy. The rationale behind IAT in patients who received EVT is twofold: first, as a salvage therapy to open residual occlusions after EVT, and second, to prevent re-occlusion because of embolization of thrombotic material into distal vessels including microcirculation (2). On the other hand, the additional administration of IAT may comprise a clinical risk of increased hemorrhagic complications that may potentially undermine its implication and even harm patients (3).

Recent trials presented at the World Stroke Conference 2024 reported promising results on the safety and efficacy of IAT with Tenecteplase and urokinase (ATTENTION IA: NCT05684172; POST UK: ChiCTR2200065617), with focus on patients with successful reperfusion following EVT.

A randomized study from Catalonia described a significantly improved functional outcome for patients with IAT. However, only half of the participants received IAT in combination with prior administration of IVT, and no safety sub-analysis of this population was presented, leaving room for controversy (4). In cases with unsuccessful reperfusion retrospective data indicated that the application of additional intraarterial thrombolysis might be safe, although only just over half of the sample received a combination of both intravenous and intraarterial thrombolysis (5).

The present study aimed to provide further evidence on the effectiveness and safety of additional IAT in patients with AIS. Patients with cerebral vessel occlusion who received IVT and EVT followed by IAT—referred to as “triple treatment” (TT)—were identified from a prospective registry. The decision to administer additional IAT was based on individual clinical judgment by the treating physician and was independent of prior reperfusion success. Outcomes of patients receiving TT were compared to a propensity score-matched control group treated with IVT and EVT alone. Hence, we intended to evaluate the safety and effectiveness of additional IAT after combined IVT and EVT treatment, focusing particularly on bleeding risk in the setting of extended thrombolytic exposure.

## 2 Methods

### 2.1 Patient selection and study design

This observational study was approved by the local ethics committee of the Justus-Liebig University Giessen, Germany (Institutional review board Nr. 220/21). We screened the Giessen Stroke Registry (GIST; ClinicalTrials.gov Identifier: NCT05295862) for patients with AIS and an occlusion of the extra- or intracranial ICA, the ACM (M1-M3) or a combination as a tandem occlusion. We included all subjects who received IVT and EVT between 05/2022 and 06/2024 and identified those patients who additionally received IAT (i.e., TT) and compared them – using 1:1 propensity-matching (see statistics)—to a control group of patients with IVT and EVT only.

### 2.2 Parameter acquisition

Clinical data were retrieved from the respective institutional database and included the following parameters: patients´ demographics, prestroke modified Rankin scale, comorbidities and National Institutes of Health Stroke Scale (NIHSS) on admission. Further, we assessed laboratory results and etiology according to TOAST criteria (6).

### 2.3 Radiologic data and parameters related to IVT, EVT, and IAT

Imaging was performed using multimodal CT including CT angiography and CT perfusion. Regarding IVT, thrombolysis was administered after the initial native CT scan using exclusively rt-PA in established dosing according to the patients´ estimated body weights (7). Regarding EVT, the neuroradiological interventionalists routinely used devices for aspiration, stent retrievers or combination devices, at the discretion of the treating interventionalist. According to institutional protocols, EVT was carried out with a maximum of 5 passes. The decision to administer additional IAT (dose of intra-arterial tr-PA was 10 mg) was not performed based on a-priori defined criteria, but rather at the discretion of the treating interventionalist on an individual basis in those patients deemed to maintain sufficient reperfusion states rather with IAT (8).

We documented baseline and follow-up imaging and procedural data, including the Alberta Stroke Program Early CT Scores (ASPECTS) as well as final reperfusion state quantified with the mTICI (modified Treatment in Cerebral Infarction) score (9).

### 2.4 Outcomes

As primary outcome we defined safety of TT, i.e., frequency of hemorrhagic complications according to the radiological classification used in the European Cooperative Acute Stroke Study (ECASS) group (10). We compared both the rates of parenchymal bleedings of classes PH1 and PH2 among the intervention and the control group. This radiological classification was chosen because the majority of patients were intubated and under sedation at the time of follow-up imaging, making a reliable clinical assessment for symptomatic intracerebral hemorrhage (sICH) not feasible. In this context, the presence of PH1 or PH2 served as a surrogate for clinical deterioration.

As secondary outcomes we defined (i) the ASPECTS upon follow-up imaging on average 21.9 h after IVT (SD: 5.3 h; range: 14.7–33.0 h) after IVT, (ii) successful recanalization rates (defined as mTICI ≥2b, and excellent reperfusion, i.e., ≥2c), (iii) 7-day mortality rates and (iv) good functional outcome, defined as modified Rankin Scale (mRS-) scores ≤ 2 on discharge. The radiological assessments were carried out by two independent raters blinded to all clinical data. In cases of initial disagreement, a consensus rating was reached.

Additionally, the mTICI scores of the TT group were evaluated descriptively to determine the extent of improvement following IAT administration.

### 2.5 Statistical analysis

Statistical analysis was performed using SPSS Version 29. To mitigate potential imbalances between treatment and control groups, propensity score matching was employed. Each of the TT-patients was matched with a control patient based on the estimated propensity score, which was calculated using the following covariates: age, NIHSS, gender, and occlusion type (M1, M2, etc.). Nearest neighbor matching was utilized, with a caliper of 0.1. Distribution of data was tested using Kolmogorov–Smirnov test. Data with normal distribution are presented as mean ± standard deviation (SD) and compared using Student’s t-Test. Data without normal distribution are presented as median and range and compared using Mann–Whitney U-test. Categorial variables were presented as frequency and percentage, comparison between groups was done using Pearson chi square or Fisher’s exact test. All statistical tests were two sided, the significance level was set at *α* = 0.05.

## 3 Results

During the study period, a total 33 patients received TT with add-on intra-arterial lysis after EVT and were then propensity score-matched in 1:1 ratio to patients received IVT plus EVT but no additional IAT. The analyzed patient cohort is presented in Table 1. Both groups were well balanced with the exception of prevalence of diabetes which was more frequent in the TT group. They showed comparable demographics and frequency of pre-existing diseases as well as similar baseline NIHSS values, laboratory results and radiological findings upon initial imaging. Although the stroke etiologies were comparable, there was a non-significant trend towards a higher frequency of large-artery atherosclerosis in the TT group.

**Table 1:** Baseline and clinical characteristics.

Characteristics	IVT+EVT (*n* = 33)	IVT+EVT+IAT (*n* = 33)	*p*-value
Female sex ^a^	19 (57.6)	21 (63.6)	0.614
Age^b^ (years)	76 (66.5–87)	75 (67–82.5)	0.501
Medical history and risk factors			
Prestroke mRS ≥ 3^a^	6 (18)	4 (12)	0.492
BMI^c^	27.1 (3.4)	26.9 (4.1)	0.828
Arterial hypertension^a^	24 (72.7)	28 (84.8)	0.228
Diabetes mellitus^a^	7 (21.2)	15 (45.5)	0.037
Hypercholesterolemia^a^	16 (48.5)	16 (48.5)	1
Abnormal kidney function^a^	11 (33.3)	8 (24.2)	0.415
Atrial fibrillation^a^	15 (45.5)	14 (42.4)	0.804
Coronary artery disease^a^	6 (18.2)	7 (21.2)	0.757
Previous ischemic stroke or TIA^a^	8 (24.2)	5 (15.2)	0.353
Admission status			
Baseline NIHSS score^c^	13 (6.4)	12.4 (7.1)	0.718
Onset to admission <4.5 h^a^	23 (69.7)	24 (72.7)	0.786
Leucocytes^c^	9.6 (3.9)	10.4 (3.6)	0.36
C-reactive protein^b^	4.4 (1.5–14.1)	3.9 (1.1–9.1)	0.676
INR^b^	1 (0.9–1.1)	1 (1–1.2)	0.114
Hemoglobin^c^	132.7 (11.5)	128.2 (18.9)	0.238
Stroke etiology (TOAST classification)^a^			0.494
Large-artery atherosclerosis	5 (15.2)	9 (27.3)	
Cardioembolism	14 (42.4)	14 (42.4)	
Other determined etiology	1 (3)	2 (6.1)	
Undetermined etiology	13 (39.4)	8 (24.2)	
Initial imaging			
Right hemisphere affected^a^	18 (54.5)	12 (36.4)	0.138
Occlusion location^a^			0.301
Internal carotid artery	4 (12.1)	7 (21.2)	
Tandem lesion	6 (18.2)	3 (9.1)	
M1 middle cerebral artery segment	12 (36.4)	10 (30.3)	
M2 middle cerebral artery segment	7 (21.2)	12 (36.4)	
M3 middle cerebral artery segment	4 (12.1)	1 (3)	
Baseline ASPECTS^b^	10 (10–10)	10 (10–10)	0.149

IVT indicates intravenous thrombolysis; EVT, endovascular thrombectomy; IAT, intra-arterial thrombolysis; mRS, modified Rankin scale; BMI, body mass index; TIA, transient ischemic attack; NIHSS, National Institutes of Health Stroke Scale; INR, international normalized ratio; TOAST, Trial of Org 10,172 in Acute Stroke Treatment; ASPECTS, Alberta Stroke Program Early CT Score. Note that no significant differences remain after correction for multiple testing.

a n (%).

b Median (IQR).

c Mean (SD).

Regarding the primary outcome, there was no significant difference between both groups (Table 2). The rate of PH1 and PH2 was 3/33 (9%) in the intervention group vs. 4/33 (12%) in the control group (OR 0.725, 95% CI 0.149–3.525, *p* = 0.689). In the IAT group, two patients suffered a class I parenchymal hemorrhage (PH1) and another patient suffered a class II hemorrhage (PH2), whereas in the control group, one patient suffered a PH1 and three patients suffered PH2 (Table 2).

**Table 2:** Analysis of primary and secondary outcomes.

Characteristics	IVT+EVT (*n* = 33)	IVT+EVT+IAT (*n* = 33)	*p*-value
Primary outcome			
PH1 + PH2 ^a^	4 (12)	3 (9)	0.689
Secondary outcomes			
Final ASPECTS^b^	8 (6–9)	8 (5.25–9.25)	0.828
Revascularization (mTICI ≥2b)^a^	30 (91)	30 (91)	0.492
7-day mortality^a^	3 (9.1)	8 (24.2)	0.099
Good functional outcome at discharge (mRS score 0–2)^a^	9 (27.3)	8 (24.2)	0.78

IVT indicates intravenous thrombolysis; EVT, endovascular thrombectomy; IAT, intra-arterial thrombolysis; ASPECTS, Alberta Stroke Program Early CT Score; mTICI, modified treatment in cerebral infarction; mRS, modified Rankin scale.

a *n (%).*

b Median (IQR).

**Figure 1:**
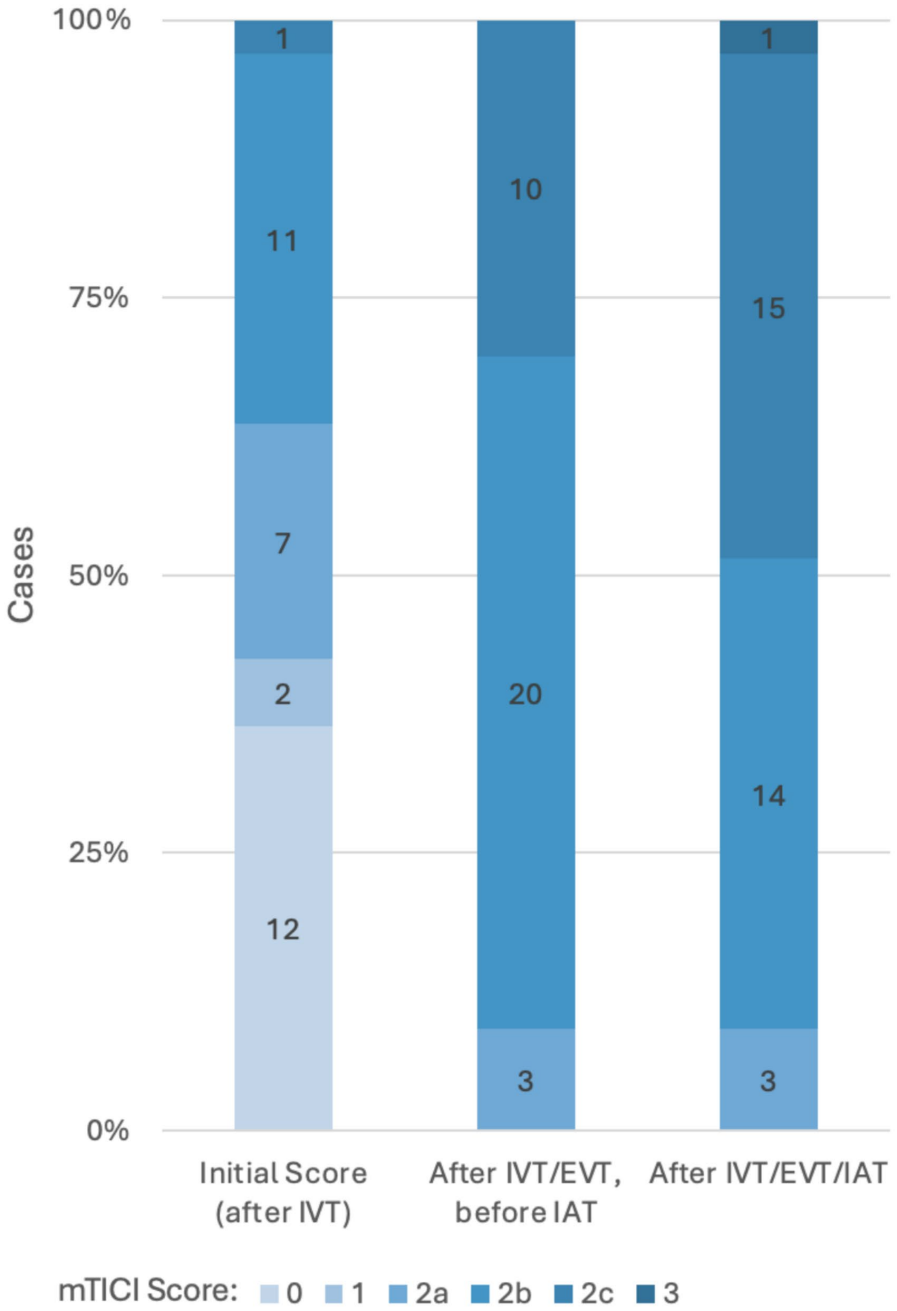
Reperfusion improvement in the group with additional IAT mTICI indicates modified treatment in cerebral infarction score; IVT, intravenous thrombolysis; EVT, endovascular thrombectomy; IAT, intra-arterial thrombolysis.

With regard to secondary outcomes, successful reperfusion (mTICI ≥ 2b) was achieved in the majority of patients without differences between both groups (Table 2). Of the 20 patients within the TT group who achieved successful reperfusion after IVT + EVT, the addition of IAT resulted in six patients achieving excellent reperfusion (mTICI ≥ 2c) after IVT + EVT and additional IAT (see Figure 1). There were no differences in the follow-up ASPECTS between the two groups, and functional outcomes at discharge were similar. There was a higher rate of 7-day mortality in the TT group however this difference was not statistically significant.

## 4 Discussion

In essence, this study found that (i) additional IAT after full-dose IVT was not linked to increased ICH, but (ii) did also not benefit the patient, neither with respect to final ASPECTS nor final mTICI grade. Some aspects deserve attention.

There is an ongoing trend in AIS treatment strategies exploring more aggressive antithrombotic management even in the hyperacute phase. This development first spilled over to stroke treatment from RCTs performed in acute coronary syndrome with focus on aggressive and early secondary prevention, e.g., dual antiplatelet therapy. For instance, the CHANCE study program and others demonstrated certain benefits of more aggressive antithrombotic treatment in AIS (11). With regard to critical situations and rescue interventions, even more risky approaches, such as acute use of glycoprotein IIb/IIIa inhibitors in the setting of rescue stenting in otherwise not recanalization able LVO have been suggested, while their clinical value appears more questionable (12, 13).

In this context, in the past months a discussion became increasingly prominent that raised the question whether IAT, administered after EVT in individuals with incomplete reperfusion states, would possibly benefit patients. Recent randomized trials were inconclusive ranging from no benefits to more favorable outcomes of TT compared to EVT alone (ATTENTION IA: NCT05684172; POST UK: ChiCTR2200065617) (4). These trials were problematic regarding analysis of safety of TT, given that only a subset of patients received IVT and no rt-PA was used for IAT. A further trial currently recruiting allows a combination of IVT and IAT, but is restricted to patients with incomplete reperfusion after EVT (NCT05499832).

Hence, the findings presented here add knowledge to the field as we, on the one hand, demonstrate no safety concerns of TT. While this finding is in line with a recent observational study (8), it appears contra-intuitively given a recommended maximum dose of rt-PA due to the increased ICH-occurrence associated with the total dose of rt-PA (14). Nonetheless, an increasing number of reports support the assumption that there is no apparent harm or increased rate of ICH from the use of TT in acute stroke patients with LVO. On the other hand, the question whether or not expanding the upper limit of rt-PA using IAT in addition to IVT and EVT is valuable, remains inconclusive considering our and recent observational data (8). Yet, in light of no signals of an increased efficacy in terms of final ASPECTS and mTICI grades, for now one should refrain from a general use of TT as long as we do not have sufficient data from RCTs proving no harm of TT.

This small study has several limitations, notably the monocentric, retrospective and non-randomized design, why residual confounding and bias by indication may have occurred. There were no standardized criteria for the decision to administer additional IAT. The low degree of tissue involvement assumed from a low initial ASPECTS suggests a highly selective patient clientele, which limits the generalizability. Furthermore, a relevant proportion of patients with medium vessel occlusion were included, meaning that the risk of bleeding complications resulting from the extent of the infarct was potentially lower than in a cohort with mainly LVO. There was a trend towards a more frequent occurrence of large-artery atherosclerosis etiology in the TT group potentially affecting results. Further, we did not assess initial and final infarct volumes using volumentrical approaches. The analysis of safety may have been biased given that we did not routinely measure the patients´ exact body weight prior to IVT. This may have resulted in under-dosing of IVT and, in cases of IAT, a total sum of rt-PA dosing not exceeding approval thresholds. Further, we did use 10 mg rt-PA for IAT in all patients regardless of the respective body weight or IVT-related rt-PA dosage applied. Further, we have focused on radiological safety features, rather than on short- and long-term clinical outcomes. Finally, another limitation refers to the matching procedure and the shortcoming of not having all clinical data of the entire crude cohort from which the matched cohort originated. Hence, a significant difference remained in the prevalence of diabetes mellitus which was accepted as the matching prioritized other covariates considered to have a stronger confounding potential.

## 5 Conclusion

TT, consisting of full-dose IVT, EVT and additional IAT, was not significantly associated with outcomes in this case–control study. While there were no signs of harm or increased rates of ICH, the small sample size undermined detecting significant clinical benefits of TT compared to IVT and EVT alone. However, in those patients with insufficient reperfusion after IVT + EVT, the additional administration of IAT may be considered on an individual basis to achieved improved mTICI scores, but TT cannot be recommended on a general basis. Randomized trials should specifically focus on robust assessments of final infarct volumes and long-term clinical outcomes.

## Data Availability

The raw data supporting the conclusions of this article will be made available by the authors, without undue reservation.
